# Bright-Field Multiplex Immunohistochemistry in Swine PCV2 and PRRSV Lymphadenopathies

**DOI:** 10.3390/ani15121682

**Published:** 2025-06-06

**Authors:** Giulia D’Annunzio, Luisa Vera Muscatello, Chiara Tugnoli, Stefano Pesaro, Andrea Luppi, Michelangelo Fiorentino, Tania Franceschini, Alessia Grillini, Gianluca Rugna, Giuseppe Sarli, Luciana Mandrioli

**Affiliations:** 1 Istituto Zooprofilattico Sperimentale della Lombardia e dell’Emilia-Romagna “Bruno Ubertini”, 25124 Brescia, Italy; giulia.dannunzio@izsler.it (G.D.); andrea.luppi@izsler.it (A.L.); gianluca.rugna@izsler.it (G.R.); 2 Dipartimento di Scienze Mediche Veterinarie, Università di Bologna, 40126 Bologna, Italy; chiara.tugnoli3@unibo.it (C.T.); giuseppe.sarli@unibo.it (G.S.); luciana.mandrioli@unibo.it (L.M.); 3 Dipartimento di Scienze Agroalimentari, Ambientali e Animali, 33100 Udine, Italy; stefano.pesaro@uniud.it; 4 Dipartimento di Scienze Mediche e Chirurgiche, Università di Bologna, 40138 Bologna, Italy; michelangelo.fiorentino@unibo.it (M.F.); tania.franceschini@ausl.bologna.it (T.F.); alessia.gri03@gmail.com (A.G.)

**Keywords:** CD3, CD20, IBA1, IHC, PCV2, PRRSV

## Abstract

Multiplex immunostaining (mIHC) allows the simultaneous detection of multiple antigenic targets within the same tissue section, providing a deeper understanding of spatial variation in cellular distribution. The aim of the present study is to apply this technique to examine the spatial variation of lymphocyte populations in swine lymph nodes during PCV2-SD and PRRSV lymphadenopathy compared with reactive lymphoid hyperplasia. A triple immunohistochemical stain with CD3, CD20 and IBA1 antibodies for the concurrent detection of T lymphocytes, B lymphocytes and macrophages, respectively, was performed. Multiplex immunohistochemistry (mIHC) revealed that, compared to reactive hyperplasia, the most significant changes in lymph node cell populations occurred in the follicles for both PCV2 and PRRSV infections. Additionally, in PCV2 cases, notable alterations were also observed in the interfollicular areas. In PCV2-affected lymph nodes, follicles not only significantly decreased in number but also showed a marked significant reduction in CD20^+^ and CD3^+^ cells. The interfollicular region in these cases also exhibited a significant reduction in CD3^+^ cells. In contrast, PRRSV-associated lymphadenopathy showed significantly increased CD20^+^ cells in the follicles, with a similar trend noted in the interfollicular region. mIHC provides more informative results on a single tissue section, thus preserving the topographical information of the tissue and allowing a comprehensive study of cellular composition, cellular functionality and cell–cell interactions, proving to be a valuable tool for studying and understanding disease dynamics.

## Simple Summary

Single-label immunohistochemistry (sIHC) is used in infectious disease studies to detect etiological agents, and to quantify and spatially localize antigens in lesions. While sIHC is limited to staining one antigen per tissue section, multiplex immunohistochemistry (mIHC) offers significantly more detailed information and is more cost- and time-efficient than repeated sIHC. Given that swine lymph node pathology involves variations in T and B lymphocytes and macrophages, this study aims to apply mIHC to visualize simultaneously and on the same slide their spatial distribution and variation across the three main regions of the swine lymph node: follicles, interfollicular areas and medulla-like tissue. We demonstrated the use of bright-field mIHC for in situ analysis of spatial cellular distribution in swine lymph nodes during PCV2-SD and PRRSV lymphadenopathy, revealing distinct lymphocyte distributions compared to reactive lymphoid hyperplasia. The study highlights the potential of mIHC as a valuable tool for understanding and studying disease dynamics.

## 1. Introduction

Single-label immunohistochemistry (sIHC) is a standard method for detecting a single protein target within a tissue section, widely employed in both diagnostic and research pathology [1]. On the other hand, multiplex immunohistochemistry (mIHC) allows the simultaneous detection of multiple protein targets on a single tissue slide, providing a more comprehensive understanding of protein/cell expression and interaction. The information derived from a single biomarker via sIHC is inherently limited, whereas the knowledge acquired from the assessment of multiple biomarkers through mIHC provides a deeper understanding of the complex interactions among these proteins and the spatial variation in cellular distribution [2]. Among mIHC techniques, brightfield multiplexing, in which antigen–antibody reactions are highlighted using chromophores, and multiplex immunofluorescence staining, which employs fluorophores together with tyramide signal amplification, are widely used [2].

Single-label immunohistochemistry (sIHC) remains the most widely employed method for investigative studies in swine pathology, particularly in the examination of lymph nodes. Investigations on these latter have a significant diagnostic value in swine pathology due to their role as secondary lymphoid organs where the adaptive immune response occurs [3]. Additionally, they are target organs for both gross and microscopic lesions caused by infectious agents such as porcine circovirus type 2 (PCV2) and porcine reproductive and respiratory syndrome virus (PRRSV). Both these viruses cause significant health problems in pig farming [4], by infecting macrophages and impairing host defenses at various levels, leading to increased susceptibility to opportunistic secondary infections. Most studies on variations in cell populations within lymph nodes during natural or experimental PCV2 [5] or PRRSV [6] infections have been conducted using single-label immunohistochemistry (sIHC). However, data on bright-field multiplex immunohistochemistry are lacking, limiting the ability to better characterize simultaneous spatial variations across different regions of the swine lymph node. PCV2 systemic disease (PCV2-SD) is characterized by severe impairment of immune system functions, resulting from lymphoid depletion and changes in the lymphocyte subpopulations [7]. Particularly in the lymph nodes, these include a severe depletion of B and T lymphocytes, an increase in the number of macrophages and a loss or redistribution of interfollicular dendritic cells [8]. The depletion of T lymphocytes mainly involves CD4+ cells and, to a lesser extent, CD8+ cells [5].

Post-natal infections with PRRSV are well documented to induce systemic lymphadenopathy and lymphocytic infiltration, predominantly within the lung [9]. Lymphoid tissues, including lymph nodes, tonsils and spleen, show apoptosis and necrosis of centrofollicular lymphocytes within germinal centers, especially during the early stages of infection, followed by follicular hyperplasia and paracortical hyperplasia in the lymph nodes during later stages [10,11,12,13]. Lymphoid depletion followed by hyperplasia is also observed in the periarteriolar lymphoid sheaths of the spleen and in the lymphoid follicles of the tonsils and Peyer’s patches [14,15].

In PCV2-induced lymphadenopathy, the reduction of B and T lymphocytes and increased macrophages are the main changes revealed by cell immunophenotyping. In contrast, in PRRSV-induced lymphoid hyperplasia, a notable increase in B cells within germinal centers is observed [16], in association with a slight increase in CD3-positive T lymphocytes [17].

Standard experimental PCV2 and PRRSV infection studies often assess residual viral load [18,19,20], but spatial cell distribution changes in lymphoid tissues are rarely investigated [5,21,22]. The availability of fully automated multiplex immunostaining systems, which enable the simultaneous detection of multiple antigenic targets within the same tissue section, has improved our ability to investigate the hypothesis that changes in the quantity or distribution of cell populations in swine lymph nodes may serve as disease-specific signatures. The aim of the present study is to examine the spatial variation of lymphocyte populations by bright-field mIHC in lymph node sections during PCV2-SD and PRRSV lymphadenopathy, compared with reactive lymphoid hyperplasia. Commercially available anti-CD3, -CD20 and -IBA1 antibodies were used for concurrent detection, respectively, of T lymphocytes, B lymphocytes and macrophages in formalin-fixed paraffin-embedded (FFPE) tissue samples.

## 2. Materials and Methods

### 2.1. Caseload

Porcine lymph nodes were initially screened and selected using hematoxylin–eosin staining and subsequently analyzed by multiplex immunohistochemistry. Screening criteria included a fixation time of no longer than 48 h to ensure optimal immunohistochemical reactivity, as well as the availability of both PCR and immunohistochemistry results for PCV2 and PRRSV. The cases that met the screening criteria included five lymph nodes with reactive lymphoid hyperplasia, which were negative for both PCV2 and PRRSV by PCR and immunohistochemistry; eight inguinal lymph nodes from eight swine diagnosed with PCV2-SD, as confirmed by both PCR and immunohistochemistry; and sixteen mediastinal lymph nodes from pigs diagnosed with PRRSV infection, with positive PCR and immunohistochemical results in lung. All tissues were available as FFPE tissue blocks. The pigs, aged between 8 and 12 weeks, came from seven wean-to-finishing sites in Northern Italy. They exhibited failure to thrive and respiratory disease and were submitted for necropsy for diagnostic purposes.

### 2.2. Triple Immunohistochemical Stain

Four-micrometer-thick sections underwent triple immunohistochemical staining using an automated immunostainer (Discovery Ultra©, Roche Diagnostics, Basel, Switzerland). Deparaffinization was achieved with EZ prep solution and antigen retrieval was performed using CC1 solution at 95° for 40 min. A triple staining protocol was employed to identify T lymphocytes, B lymphocytes and macrophages, using CD3 (Roche; Basel, Switzerland, clone 2GV6), CD20 (Invitrogen; Waltham, MA, USA, polyclonal rabbit, 1:300 dilution) and a polyclonal antibody against IBA1 (Novus; Cambridge, UK, goat polyclonal, 1:2500 dilution) as primary antibodies. CD3 was dispensed and incubated for 32 min at 36°, the slides were treated with rabbit multimer (Rb-HRP) and subsequently with DAB (diaminobenzidine) for detection of the reaction using the Discovery ChromoMap DAB Kit (code n. 05266645001, Roche Diagnostics, Basel, Switzerland). This kit consists of three different dispensers. “DISCOVERY DAB CM” contains phosphate buffer, diaminobenzidine (DAB) and gentamicin sulfate. It is applied for 8 min. “DISCOVERY H_2_O_2_ CM” contains phosphate buffer and hydrogen peroxide (H_2_O_2_). It was applied for 4 min. “DISCOVERY Copper CM” contains an acetate buffer, cupric sulfate and gentamicin sulfate. It was applied for 4 min.

IBA1 was dispensed, incubated for 24 min at 36° and amplified using Amp A and B. Subsequent to treatment with mouse multimer (Rb-HRP), sections were processed with the Discovery Purple HRP kit (code n. 07053983001, Roche Diagnostics, Basel, Switzerland). The latter is made of two different dispensers. “DISCOVERY Purple” contains a purple chromogen; it was applied for 4 min, and “DISCOVERY H_2_O_2_” contains hydrogen peroxide in an aqueous solution; it was applied for 32 min.

For the detection of CD20, we proceeded with “option diluent” that blocks nonspecific binding sites. The antibody was dispensed, incubated for 36 min at 36 °C and amplified (Amp A and B). Rabbit multimer (Rb-HRP) served as the secondary antibody, followed by the Discovery Teal HRP staining kit (code n. 08254338001, Roche Diagnostics, Basel, Switzerland). This kit consists of three dispensers. “DISCOVERY Teal HRP H_2_O_2_” contains a borate buffer and hydrogen peroxide, it is applied for 4 min. “DISCOVERY Teal HRP Substrate” contains a borate buffer and a reactant to a component in the Teal Activator; it was applied for 8 min. “DISCOVERY Teal HRP Activator” contains a borate buffer and a reactant to a component in the Teal Substrate; it was applied for 8 min.

Finally, the slides were washed with Reaction Buffer.

In the staining procedure was also used the “Amplification Kit” (Amp A and B) and the Rb-HRP (anti-rabbit HRP). Amplification Kit (code n. 05266114001, Roche Diagnostics, Basel, Switzerland) utilizes rabbit anti-mouse IgG heavy and light chains and mouse anti-rabbit IgG heavy chains to bind to the primary antibody, increasing the number of antibodies bound at the site of antigen and increasing the staining intensity. Both reagents are applied for 8 min. Rb-HRP (code n. 05269717001, Roche Diagnostics, Basel, Switzerland) is a biotin-free detection system based on proprietary multimer technology. They consist of a robust chemistry that provides a clean background in combination with enhanced specificity and sensitivity, which increases the signal-to-noise ratio.

The specificity of the immunohistochemical stain has been validated by adding, as the primary reagent, an aspecific antibody of the same isotype as the primary antibodies.

### 2.3. Image Analysis

For each group (reactive hyperplasia, PRRSV and PCV2 lymphadenopathy), three immunostained sections were selected for image analysis to quantify the proportion of tissue area occupied by each cell population, based on the chromogenic signal of the immunohistochemical staining.

Immunohistochemically stained slides were digitized using the Grundium Ocus 20 scanner (Tampere, Finland) to obtain whole-slide images (WSIs). Advanced image analysis was then performed using QuPath 0.5.1 software.

A standardized lymph node tissue area of 17,000,000 µm^2^ was selected for each case to evaluate follicles, the interfollicular area and medulla-like tissue. Within this area, the number of follicles was determined using the “point” tool. Subregions corresponding to the follicles, interfollicular area and medulla-like tissue were then delineated, and within each subregion, the quantification of CD20^+^, CD3^+^ and IBA-1^+^ immune cell labeling was performed.

Immune cell quantification was achieved using pixel classification tools, which enable area-based analysis of labeled cells through chromatic recognition. Classifiers for CD20^+^, CD3^+^ and IBA-1^+^ cells were developed using the Random Tree algorithm with high spatial resolution (0.50 µm/px). The training involved an average of 25 manual annotations per marker, performed by an experienced QuPath user (C.T.) who was blinded to the group assignments.

T and B lymphocyte and histiocyte measurements were expressed as the percentage of area covered within each targeted region.

## 3. Results

According to histologic descriptions of swine lymph nodes [5,8], three main areas of cellular localization were defined: (1) B cell zones, which include primary and secondary follicles, (2) T cell territories, located within the interfollicular area and (3) the sub-capsular medulla-like tissue. The application of the mIHC technique allowed the concurrent visualization of CD3+ cells (T lymphocytes) stained brown with diaminobenzidine (DAB), CD20+ lymphocytes (B lymphocytes) stained blue with Discovery Teal HRP, and IBA1+ histiocytes/macrophages, stained fuchsia using Discovery Purple HRP. Positive staining was observed in the cell membrane for CD3 and CD20 antigens, and in the cytoplasm of IBA1-positive cells.

**Figure 1:**
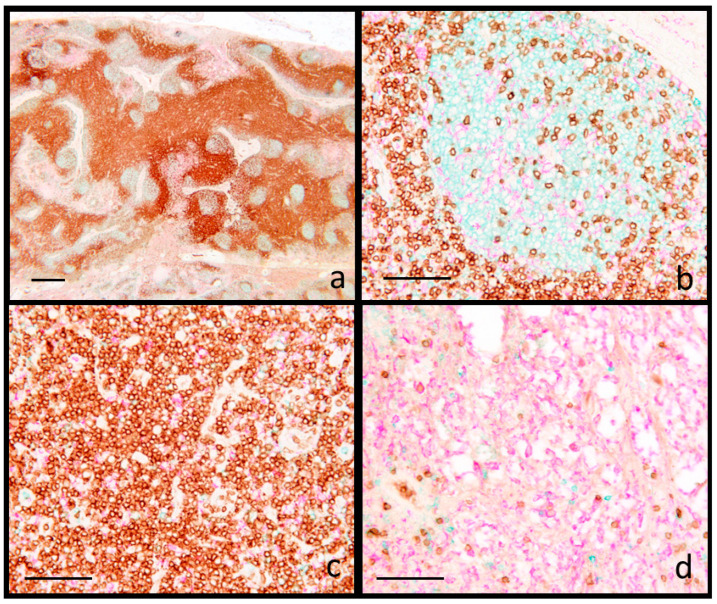
Swine. Inguinal lymph node. Reactive hyperplasia. Multiplex immunohistochemistry for CD3 (brown), CD20 (blue) and IBA1 (fuchsia). (**a**) Multiple reactive follicles (higher magnification in (**b**) containing mainly CD20+ lymphocytes with scattered CD3 lymphocytes and IBA1+ cells; (**c**) interfollicular areas containing CD3+ lymphocytes and few IBA1+ cells; (**d**) the medulla-like tissue shows a prevalence of IBA1+ reticular cells. Scale bar: (**a**) 800 μm; (**b**–**d**) 100 μm.

Lymphoid hyperplasia was characterized by secondary follicles with prominent germinal centers and expanded interfollicular area, accompanied by a diminution in the extent of the medulla-like tissue (Figure S1a–c). The spatial distribution of cells showed strict compartmentalization: CD20+ cells were localized mainly within the germinal centers (B cell areas), associated with scant CD3+ and IBA1+ cells; interfollicular (T cell) areas were populated mainly by CD3+ and few IBA1+ cells, while the majority of IBA1+ cells were present in the medulla-like tissue (Figure 1).

PCV2-SD cases, all confirmed positive for PCV2 by sIHC, were characterized by moderate to severe lymphoid depletion [23], ranging from follicle reduction/absence (five cases out of eight) to reduced lymphocytes density in interfollicular areas (seven cases out of eight). Concomitantly, epithelioid and giant cells were detected within both follicular and interfollicular areas (Figure S1d–f), thus fulfilling the current diagnostic criteria for PCV2 diseases [24].

**Figure 2:**
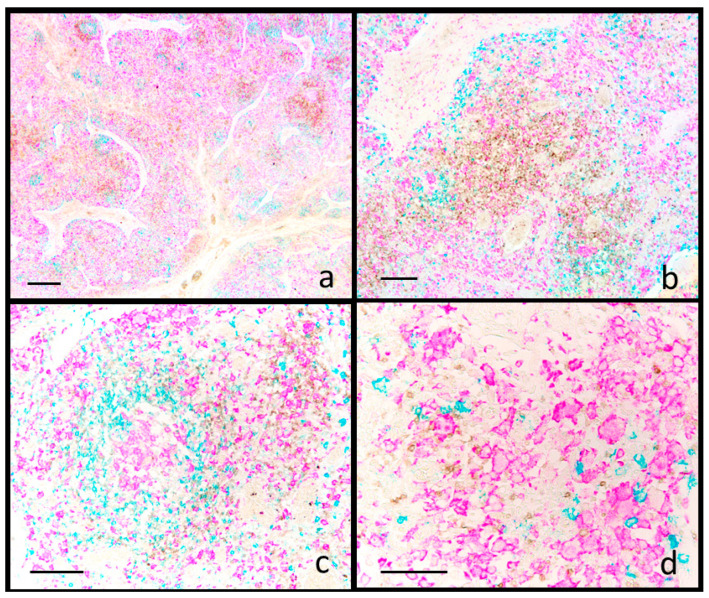
Swine. Inguinal lymph node. PCV2 systemic infection. Multiplex immunohistochemistry for CD3 (brown), CD20 (blue) and IBA1 (fuchsia). (**a**) Sparse and fading follicles show a reduction of CD20+ lymphocytes (**b**) and the presence of IBA1+ follicular epithelioid cells (**c**). In the interfollicular area, CD3+ lymphocytes are severely reduced (**b**,**c**), while IBA1+ macrophages are increased (**b**). IBA1 staining is also detectable in epithelioid and giant cells (**d**). Scale bar: (**a**) 800 μm; (**b**) 200 μm; (**c**) 150 μm; (**d**) 100 μm.

Quantitative data from image analysis are presented in Figures S2 and S3. Compared to reactive hyperplasia, PCV2 cases showed a significant reduction in the number of follicles (Figure S2a), as well as a significant decrease in CD20^+^ (Figure S2b) and CD3^+^ lymphocytes (Figure S2c) within the follicles. Although not statistically significant, in the same compartment, there was a trend toward increased IBA1^+^ macrophages (Figure S2d). In the interfollicular area, the only significant change observed was a reduction in CD3^+^ cells (Figure S3b). Despite these quantitative changes (CD20^+^ and CD3^+^ cells were reduced, while IBA1^+^ cells tended to increase), the overall localization of immune cells was preserved in PCV2 cases. In the follicle remnants, CD20^+^ B cells and IBA1^+^ macrophages predominated, whereas the interfollicular areas exhibited variable numbers of CD3^+^ T cells alongside an increased presence of IBA1^+^ macrophages (Figure 2).

**Figure 3:**
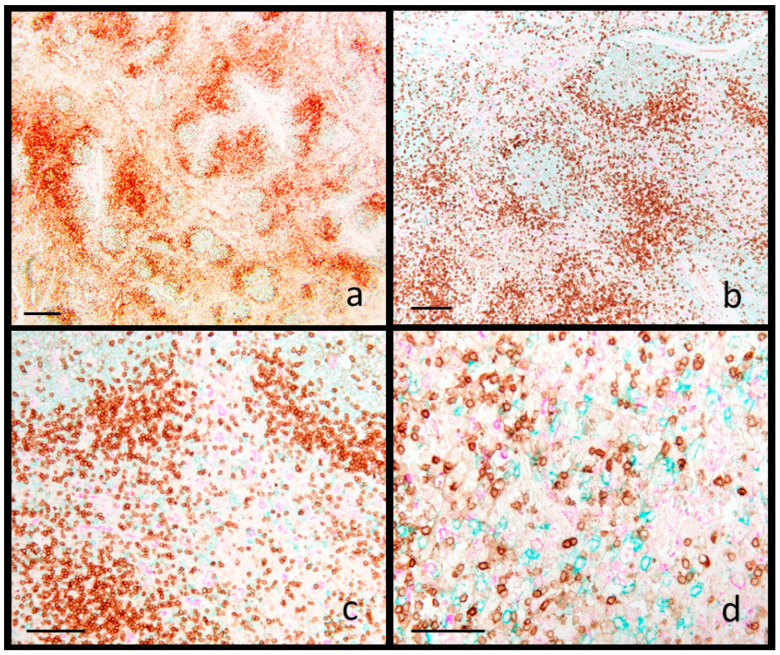
Swine. Mediastinal lymph node. PRRSV infection. Multiplex immunohistochemistry for CD3 (brown), CD20 (blue) and IBA1 (fuchsia). (**a**) Multiple reactive follicles rich in CD20+ lymphocytes (higher magnification in (**b**). (**b**–**d**) The interfollicular area shows an increase of CD20+ lymphocytes, associated with a decrease of CD3+ lymphocytes. Scale bar: (**a**) 800 μm; (**b**) 200 μm; (**c**) 150 μm; (**d**) 100 μm.

PRRSV-positive cases, confirmed positive by sIHC in lung and by PCR in a pool of lung and lymphoid tissues, revealed severe hyperplasia of the interfollicular areas, characterized by the expansion of blast cells (13 cases out of 16) with scattered small lymphocytes (Figure S1g,h). Multifocal karyorrhexis within lymphoid tissues was observed in six cases (Figure S1i). Immunophenotypic analysis, performed by the mIHC staining methodology, revealed an expansion of CD20+ cells within germinal centers (12 cases out of 16) (Figure 3) and also within the interfollicular area (10 cases out of 16) (Figure 3). These interfollicular areas were characterized by a predominance of CD20+ blast cells interspersed with small CD3+ lymphocytes. In Figures S2 and S3, the quantitative data from image analysis are reported. Compared to reactive hyperplasia, PRRSV cases show a similar number of follicles (Figure S2e), but not invariate cell-type predominance (CD20+) in follicles that exhibited a significant increase of the CD20+ area (Figure S2f). Other changes detected by quantitative analysis are trends to an increase of CD20+ cells and a decrease of CD3+ cells in the interfollicular area (Figure S3d,e). The net result of PRRSV lymphadenopathy is an expansion of B cells, mainly in follicles and to a lesser extent in the interfollicular area.

Medulla-like tissue was the only compartment in which no significant variation in cell distribution was observed in either PCV2 or PRRSV cases. However, a trend toward a reduction in both CD20^+^ and CD3^+^ cells was more evident in PCV2 cases compared to PRRSV (Figure S4).

## 4. Discussion

Histopathology is crucial for the diagnosis of infectious diseases in pigs, as it enables the identification of lesion patterns suggestive of specific pathogens. For example, cell depletion and granulomas in swine lymphoid tissues are indicative of PCV2 systemic disease [24,25]. Additionally, highly specific techniques like immunohistochemistry (IHC) and in situ hybridization (ISH) establish disease causality by linking specific antigens or nucleic acids present to characteristic lesions [26,27,28].

While histopathology and pathogen co-localization with lesions are gold standards for diagnosing some swine infectious diseases [24,25], the value of these in situ methods is undeniable for studying pathogen roles in major pig diseases [29,30,31,32], co-infection models [33,34,35,36] and in pathogenesis studies [26]. Although sIHC remains a widely used technique, the application of mIHC provides significantly more detailed information and confers the advantage of cost-effectiveness and time efficiency compared to performing multiple rounds of sIHC [2]. Furthermore, while automated mIHC has markedly improved efficiency and standardization compared to traditional staining methodologies, the manual handling of numerous slides and the subsequent examination and analysis of stained sections continue to be labor-intensive processes [2].

Bright-field mIHC is preferred for visualizing the presence and amount of different cell types in tissue sections, and it can be performed on archival FFPE tissues. Furthermore, compared to multiplex immunofluorescence, it eliminates the need for UV microscopes and avoids signal fading over time. Platforms for mIHC, exemplified using the Discovery Ultra© instrument (Roche Diagnostics, Basel, Switzerland) used in this study, allow complex assay development performing multiple biomarkers analysis using a wide range of new chromogenic dyes. This facilitates in situ analysis with conventional brightfield microscopes. These chromogens have broad absorbance spectra which produce dark staining patterns that are supposedly easy to distinguish by light microscopy [37]. More importantly, conventional scanners can acquire images of these stained slides, enabling biomarker quantitation.

The host–pathogen interaction is a dynamic process, initiating with the innate immune response followed by the adaptive response to resolve infections. In germinal centers of secondary lymphoid tissues, such as lymph nodes, antigen presentation by dendritic cells (antigen-presenting cells) triggers antigen-dependent differentiation of antigen-specific T and B lymphocytes along with antibody isotype switching and affinity maturation. This culminates in the development of a targeted and robust immune response capable of effectively resolving the infection [38]. Thus, the use of individual immunohistochemical markers to characterize lymph node responses has been applied in research to expand knowledge related to the pathogenesis and modulation of immune response. Indeed, there is evidence that immunosuppressive pathogens, such as PCV2, induce alterations (absolute or relative) in the proportions of different lymphocyte subsets during natural infection. These changes appear to correlate with PCV2 levels in lymphoid tissues and with the degree of depletion observed in the B- and T-cell-dependent areas of these tissues [39].

Since swine lymph node pathology studies concern variations of the main cell populations, namely T and B lymphocytes and macrophages, the primary goal of this study was to apply mIHC stain to these cell types in order to obtain, on a single slide, an indication of their spatial distribution and variation within the three principal anatomic regions of the swine lymph node: follicles, interfollicular areas and medulla-like tissue. When present, variations in immune cell populations may contribute to explaining the efficacy of the immune response. The humoral response is predominantly associated with the expansion of CD20+ B lymphocytes, whereas the cell-mediated response primarily involves the function of CD3+ T lymphocytes [40]. Both branches of the adaptive immune system can be evaluated in the context of spontaneous or experimentally induced diseases, as well as following vaccination, using serological methods or in vitro assays. Likewise, histological analysis—through the identification of specific cell types and their spatial distribution—serves as a valuable tool for assessing immune dynamics in these contexts.

Most studies on lymphoid tissues during PCV2 or PRRSV infections describe variations in cell types using semiquantitative estimations [5,6]. However, there is a recognized need for truly quantitative and objective data to allow for meaningful comparisons across studies. Manual biomarker quantification is discouraged due to its subjective nature and lack of reproducibility [41]. Instead, software like QuPath (0.5.1), which uses machine learning-based digital analysis, allows the evaluation of entire tissue sections, reducing the subjectivity involved in area selection. This approach produces objective and reproducible data from whole-section analyses, enabling the collection of high-quality information [41]. The data presented in the Supplementary Materials provide a numerical assessment of increases or reductions in PCV2 or PRRSV cases, in comparison with reactive hyperplasia, along with the statistical significance—or lack thereof—supporting the observed results. We acknowledge that the limited number of cases included in the image analysis represents a constraint of the present study, and that additional cases are needed to confirm and strengthen our preliminary findings.

Histologic lesions observed in lymphoid tissues during PCV-SD have been characterized using sIHC methods [5,6,7,8,42], and mainly consist of a reduction in B cells, depletion of T-dependent areas, an increase in subcapsular and peritrabecular macrophages, and partial loss and redistribution of antigen-presenting cells in lymphoid tissues [8]. The severity of variations related to lymphocyte subpopulations is strongly correlated with both the severity of the histological lesions and the amount of PCV2 antigen or nucleic acid present within the lesions [21]. The lymphocyte population exhibiting the most significant depletion is recognized to include CD4+ T helper cells, with CD8+ cytotoxic T cells being affected to a lesser extent [5]. In our study, the triple immunohistochemical method, designed for the simultaneous detection of CD3+ (T lymphocytes), CD20+ (B lymphocytes) and IBA1+ (macrophages) cells was applied on a single tissue section. It allowed us to study the spatial variation of lymphocyte populations, highlighting a moderate/severe reduction of both B and T lymphocytes in two of the three areas of the swine lymph node: follicles and the interfollicular area.

In PRRSV^+^ cases, the observed lymphadenopathy was characterized by follicular hyperplasia and multifocal necrosis within the interfollicular areas, accompanied by an expansion of immature blast cells. Phenotypic analysis revealed CD20^+^ cells not only within follicles but also increased in the interfollicular area. This finding contrasts with the typical pattern of PRRSV-induced lymphoid hyperplasia, in which T lymphocytes usually predominate in the interfollicular areas [43], although B cell expansion has also been reported in such lymphadenopathy. One speculative interpretation of this B cell expansion comes from Lamontagne et al. [44], who suggested that PRRSV infection induces a polyclonal expansion of B cells within follicles, potentially driven by signals from persistently infected macrophages. An alternative hypothesis posits an impact on B cell maturation [45,46]. Notably, PRRSV infection impairs thymic function and disrupts signaling between follicular helper T cells and B cells, resulting in immunosuppression—primarily through interference with the development of an effective innate immune response [46]. The presence of a true increase in B cells during experimental PRRSV infection has been documented by Lamontagne et al. [44] in the spleen and tonsils, and by Kawashima et al. [47] in lymph nodes. In our study, the detection of CD20^+^ blast cells in the interfollicular areas was observed in ten out of sixteen cases, indicating that further confirmation in a larger cohort is needed.

In addition to the limited number of cases selected—based on the availability of PCR and sIHC tests for PCV2 or PRRSV, as described in the Materials and Methods section—other limitations should also be acknowledged due to the novelty of the method employed. One key consideration is the steric hindrance, in which antibodies compete for binding while labeling markers in the same cell type. This is a well-known issue in multiplex immunohistochemistry (mIHC), particularly when different stains target the same cell type and subcellular compartment [48] (e.g., nucleus, cytoplasm or membrane), which may lead to staining artifacts or reduced signal clarity. However, in our mIHC protocol, the antibodies used target distinct cell types, and cross-interference between staining steps appears to be minimal, thus not representing a major limitation. While mIHC is valuable for detecting spatial variations in immune cell distribution, it does not provide information on functional cell activity or cytokine production. These functional aspects would require complementary approaches to gain a more comprehensive understanding of the immune response.

## 5. Conclusions

For many years, sIHC has been employed in the study of infectious diseases as a method for detecting and distinguishing between etiological and incidental agents, as well as for quantifying and spatial localizing antigens within lesions [49]. However, one of the limitations of sIHC is its inherent restriction to the staining of a single antigen per tissue section. The detection of multiple tissue antigens may be achieved either through the performance of sIHC individually for each target antigen on serial sections or through the simultaneous application of mIHC on a single tissue section, as exemplified by the triple immunohistochemical reaction used in this study for the examinations of lymphoid cell populations within porcine lymph nodes. Compared to reactive hyperplasia, PCV2 lymphadenopathy was characterized by a decrease in CD20+ and CD3+ cells, coupled with an increase in IBA1+ macrophages, within the B and T cell areas. In PRRSV-associated lymphadenopathy, as well as the increase of CD20+ cells in follicles, a change that needs confirmation in a larger caseload, is the increased presence of CD20+ cells in the interfollicular area.

This type of multiplex immunohistochemical method provides more informative results by simultaneously detecting multiple markers on a single tissue section. This approach preserves the tissue’s topographical context and enables a comprehensive analysis of cellular composition and cell–cell interactions [37,50]. It has proven to be a valuable tool for studying and understanding disease dynamics and can be applied under both spontaneous and experimental conditions, as well as following vaccination challenges.

## Data Availability

The original contributions presented in this study are included in the article and in the supplementary material. Further inquiries can be directed to the corresponding author.

## Supplementary Materials

The following supporting information can be downloaded at https://www.mdpi.com/article/10.3390/ani15121682/s1, Figure S1: Swine lymph node. (a,b) moderate reactive hyperplasia in (a) and severe reactive hyperplasia in (b). Note the reactive follicles and the expansion of the interfollicular tissue over the medulla-like tissue, which appears reduced in b) compared to a). The 5 cases in the present study exhibited features consistent with (b). (c,d) PCV2-associated lymphadenopathy. Absence of follicles and depletion of the interfollicular tissue (c). In (d), epithelioid and giant cells are present on the right with-in a follicle remnant and on the left in the interfollicular tissue. (e,f) PRRSV-associated lymphadenopathy. Hyperplasia of the lymphoid tissue (e) associated with follicle hyperplasia (f) and interfollicular expansion by lymphoblasts (f and inset). Scale bar: (a,b,c,e) 800 μm; (d,f) 150 μm. Figure S2: Comparison of image analysis data between reactive hyperplasia (RH) and PCV2 or PRRSV. Quantitative analysis includes: number of follicles (a,e) and area (expressed as percentage) within follicles occupied by CD20^+^ (b,f), CD3+ (c,g) or IBA1+ (d,h) cells. Statistical analysis was performed using Student’s *t*-test for unpaired data. Significance: *p* < 0.05 (*), *p* < 0.001 (**). Figure S3: Comparison of image analysis data between reactive hyperplasia (RH) and PCV2 or PRRSV. Quantitative analysis includes the area (expressed as percentage) of interfollucular lymphoid tissue occupied by CD20^+^ (a,d), CD3+ (b,e) or IBA1+ (c,f) cells. Statistical analysis was performed using Student’s *t*-test for unpaired data. Significance: *p* < 0.05 (*), *p* < 0.001 (**). Figure S4: Comparison of image analysis data between reactive hyperplasia (RH) and PCV2 or PRRSV. Quantitative analysis includes the area (expressed as percentage) of medulla-like tissue occupied by CD20^+^ (a,d), CD3+ (b,e) or IBA1+ (c,f) cells. Statistical analysis was performed using Student’s *t*-test for unpaired data. Significance: *p* < 0.05 (*), *p* < 0.001 (**).

## Abbreviations

The following abbreviations are used in this manuscript:

sIHC	Single-label immunohistochemistry
mIHC	Multiplex immunohistochemistry
PCV2	Porcine circovirus type 2
PRRSV	Porcine reproductive and respiratory syndrome virus
PCV2-SD	Porcine circovirus type 2-systemic disease
FFPE	Formalin-fixed paraffin-embedded
