# Supplementary material for: Bright-Field Multiplex Immunohistochemistry in Swine PCV2 and PRRSV Lymphadenopathies
Source: Animals (Basel). 2025 Jun 6;15(12):1682. doi: 10.3390/ani15121682 (PMC12189695; doi:10.3390/ani15121682)
Supplement: Supplementary file 1 [file animals-15-01682-s001.zip › Supplementary Figure S1.pdf]

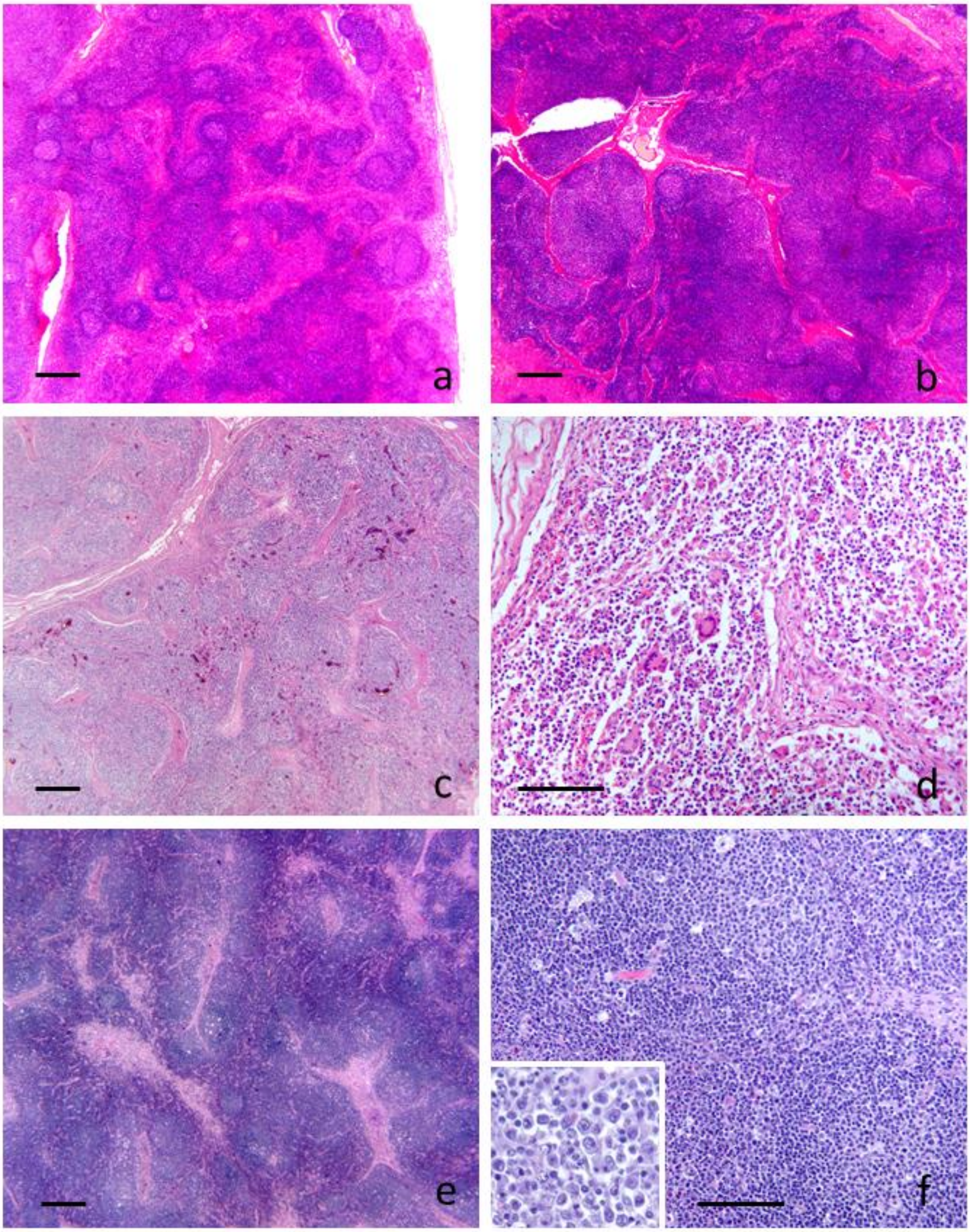

Supplementary Figure S1. Swine lymph node. a, b) moderate reactive hyperplasia in (a) and severe reactive hyperplasia in (b). Note the reactive follicles and the expansion of the interfollicular tissue over the medulla-like tissue, which appears reduced in b) compared to a). The 5 cases in the present study exhibited features consistent with b). c, d) PCV2-associated lymphadenopathy. Absence of follicles and depletion of the interfollicular tissue (c). In d), epithelioid and giant cells are present on the right with-in a follicle remnant and on the left in the interfollicular tissue. e, f) PRRSV-associated lymphadenopathy. Hyperplasia of the lymphoid tissue (e) associated with follicle hyperplasia (f) and interfollicular expansion by lymphoblasts (f and inset). Scale bar.: a, b, c, e) 800 µm; d, f) 150 µm.
