# Supplementary material for: Bright-Field Multiplex Immunohistochemistry in Swine PCV2 and PRRSV Lymphadenopathies
Source: Animals (Basel). 2025 Jun 6;15(12):1682. doi: 10.3390/ani15121682 (PMC12189695; doi:10.3390/ani15121682)
Supplement: Supplementary file 1 [file animals-15-01682-s001.zip › Supplementary Figure S3.pdf]

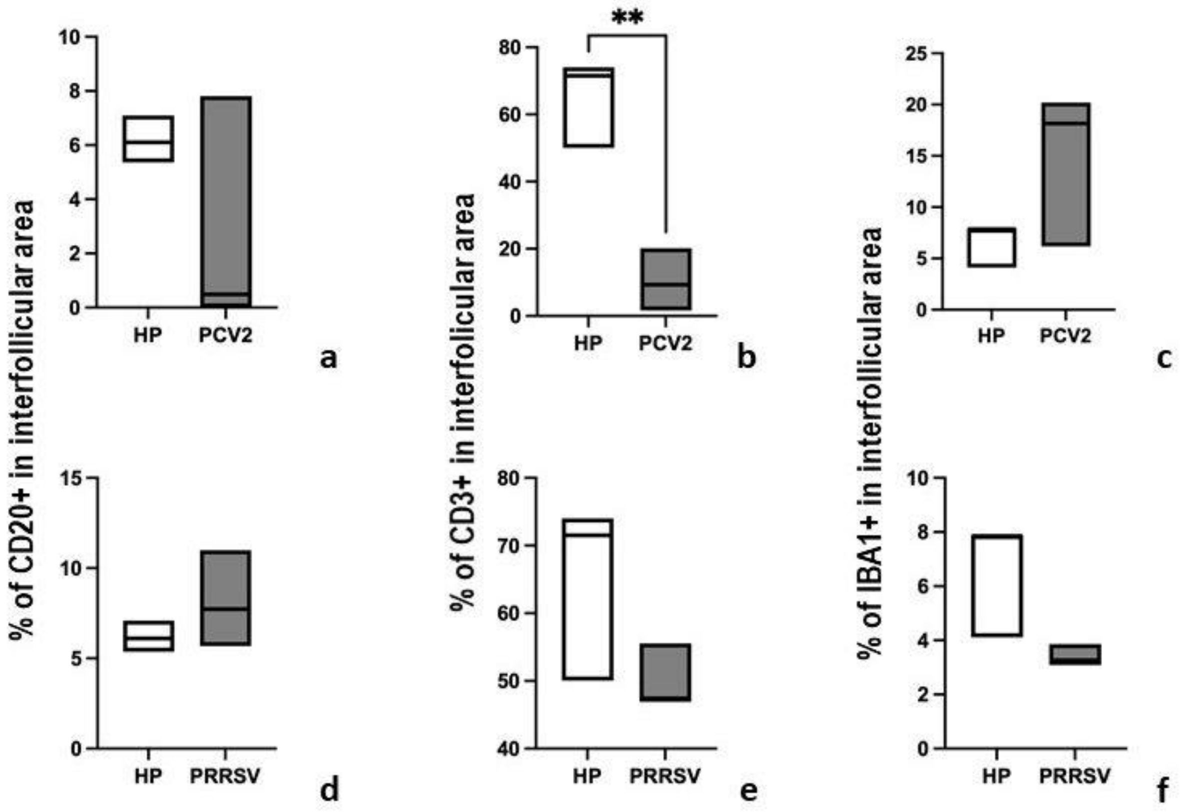

Supplementary Figure S3. Comparison of image analysis data between reactive hyperplasia (RH) and PCV2 or PRRSV. Quantitative analysis includes the area (expressed as percentage) of interfollucular lymphoid tissue occupied by $CD20^{+}$ (a, d), CD3+ (b, e) or IBA1+ (c, f) cells. Statistical analysis was performed using Student's t-test for unpaired data. Significance: $p < 0.05$ (*), $p < 0.001$ (**).
